# Adherence to multi-target stool DNA testing for colorectal cancer screening in the United States

**DOI:** 10.1007/s00384-025-04805-0

**Published:** 2025-01-17

**Authors:** Quang A. Le, Mallik Greene, Shrey Gohil, A. Burak Ozbay, Michael Dore, A. Mark Fendrick, Paul Limburg

**Affiliations:** 1 Exact Sciences Corporation, Madison, WI USA; 2 Department of Medicine, Duke University, Durham, NC USA; 3 Division of General Medicine, Department of Internal Medicine, University of Michigan, Ann Arbor, MI USA

**Keywords:** Colorectal cancer, CRC screening, Multi-target stool DNA, Screening adherence, Early detection

## Abstract

**Purpose:**

Colorectal cancer (CRC) is the second leading cause of cancer mortality in the USA and is highly preventable, with early screening vital for improving outcomes. This study aimed to evaluate adherence rates of multi-target stool DNA (mt-sDNA) testing, following updated guidelines recommending screening starting at age 45.

**Methods:**

This retrospective cohort study used aggregated data from Exact Sciences Laboratories LLC, examining new users (first-time testers) aged 45–85 with commercial, Medicare, or Medicaid insurance who received mt-sDNA test kits (point-of-care) between January 1, 2023, and June 1, 2023. Adherence was defined as the percentage of eligible participants returning a valid non-empty test kit within 365 days of initial shipment date. Descriptive statistics and logistic regression were used to analyze adherence.

**Results:**

Among 1,557,915 patients, the overall adherence rate to mt-sDNA testing was 71.3% (commercial insurance 72.3%, Medicare Advantage 70.2%, Medicare 69.9%, Medicaid 52.0%) (*p* < 0.001). Females had slightly higher adherence than males, except for commercial insurance (72.2% vs. 72.6%, *p* < 0.001). Adherence was highest in commercial insurance for individuals aged 76–85 (79.2%, *p* < 0.001), gastroenterology patients (82.5%, *p* < 0.001), and rural residents (73.2%, *p* < 0.001), along with those in Medicare Advantage earning $200 K + (78.5%, *p* < 0.001).

**Conclusions:**

Adherence to mt-sDNA testing was robust, particularly among individuals with commercial insurance, older adults, gastroenterology patients, higher income groups, and rural residents. With a 71% adherence rate, the test demonstrates substantial engagement and value in colorectal cancer screening. Future research should assess its long-term impact and address disparities to optimize its benefits.

**Supplementary Information:**

The online version contains supplementary material available at 10.1007/s00384-025-04805-0.

## Introduction

Colorectal cancer (CRC) remains a formidable public health challenge in the USA, ranking as the second leading cause of cancer-related mortality and the third most common cancer [1, 2]. Effective CRC screening programs are pivotal in mitigating this burden by identifying premalignant or localized malignant neoplasia for early intervention, thereby improving patient outcomes and reducing mortality rates [3–5].

In response to the imperative to enhance participation, national organizations have recently updated their average-risk CRC screening recommendations. The United States Preventive Services Task Force (USPSTF) now advocates for CRC screening to begin at age 45, aiming to facilitate earlier detection and prevention [4]. This adjustment marks a shift from previous guidelines, which recommended screening to commence at age 50 [6]. Despite this change, the current national uptake of CRC screening remains suboptimal and falls below the 80% goal of the American Cancer Society National Colorectal Cancer Roundtable [7]. To address this challenge, the Community Preventive Services Task Force (CPSTF) recommends implementing multi-component interventions to promote CRC screening [8]. These interventions involve employing two or more strategies to increase community demand, improve access, enhance provider recommendations, and reduce structural barriers to screening.

Patient navigation services, including patient reminders and education, have emerged as valuable tools to improve CRC screening completion [9–13]. These services have been incorporated into all multi-target stool DNA (mt-sDNA; Cologuard®) test orders, which offer an at-home CRC screening option for average-risk individuals to support patients throughout the screening process. An earlier study reported an overall adherence rate of 66.8% for mt-sDNA testing among both commercially and Medicare-insured patients [14]. However, with the recent updates to USPSTF recommendations for CRC screening and the implementation of new patient navigation services, there is a pressing need to reassess real-world adherence to mt-sDNA testing.

This study aimed to address this gap by examining the adherence rate to mt-sDNA testing among new users across different health payors and subgroups. This analysis also explored sociodemographic factors associated with patient adherence to mt-sDNA screening, which can inform strategies to enhance CRC screening uptake and reduce the burden of CRC in the USA.

## Methods

### Data source

In this study, aggregated laboratory data from Exact Sciences Laboratories LLC (ESL), headquartered in Madison, WI, the exclusive national laboratory for mt-sDNA testing, were analyzed retrospectively as a part of continuous laboratory quality management procedures and in adherence to the Health Insurance Portability and Accountability Act (HIPAA). The study was conducted in accordance with the Strengthening the Reporting of Observational Studies in Epidemiology (STROBE) guidelines for observational research. The study was exempt from institutional review board (IRB) approval, as it used deidentified aggregate data.

### Study population and design

This retrospective cohort study included individuals aged 45 to 85, covered by commercial insurance, Medicare, or Medicaid, who received the mt-sDNA test kit via a point-of-care order for the first time (new users) from ESL between January 1, 2023, and June 1, 2023. Deidentified data on patient (sex, age), provider (specialty, practice location), and test order details (testing status, completion time) were sourced from ESL’s internal systems. Data on patient income were gathered at the residential zip code level from public sources like the US Census and American Community Surveys database.

### Exclusion criteria

The analysis excluded individuals who were not new (first-time users) to mt-sDNA testing, who were not covered by commercial, Medicare, Medicare Advantage, or Medicaid insurance plans, who were aged below 45 or above 85 years, whose orders were not point-of-care, who had canceled orders other than those that expired, or who had major missing information on important patient characteristics.

### Outcome of interest

The primary outcome of interest was adherence to the mt-sDNA test, defined as the percentage of eligible participants successfully returning a valid non-empty test kit within 365 days of initial shipment date. A valid mt-sDNA test was defined as the one having all the information required for ESL to analyze the sample and report a positive or negative test result.

### Statistical analysis

Descriptive statistics were used to summarize baseline characteristics overall and by payor type, presented as frequencies, percentages, and chi-square test *p* values. Adherence was reported along with corresponding descriptive statistics and chi-square test results. The mean and standard deviation (SD) of the time to adherence, measured in days, were also reported, with *p* values derived from analysis of variance (ANOVA). Logistic regression analysis was employed to examine factors associated with adherence. The independent variables included age, sex, race, ethnicity, urban/rural classification, type of payor, preferred language, provider specialty, and median annual household income by zip code. All analyses were performed using R version 4.4.1.

## Results

**Fig. 1:**
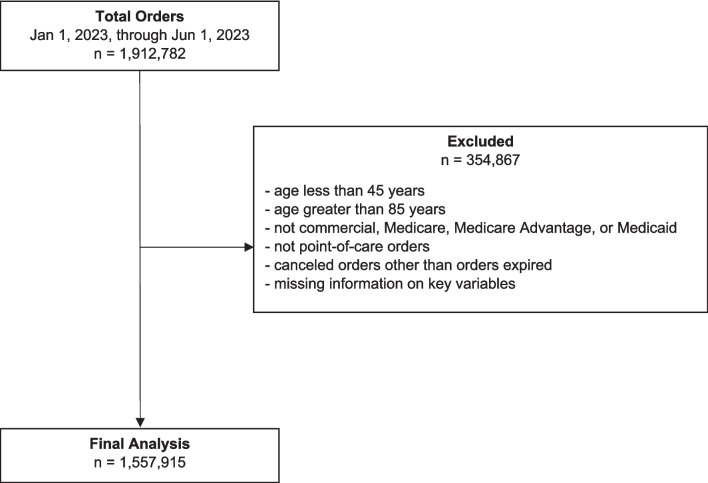
Study attrition chart

There were 1,557,915 patients who met the study criteria (Fig. 1), out of which 68.1% had commercial insurance, 21.4% had Medicare Advantage, 8.9% had traditional Medicare, and 1.7% had Medicaid. Females constituted the majority of patients across all payor categories: 57.7% in commercial insurance, 58.5% in Medicare Advantage, 59.2% in Medicaid, and 57.4% in traditional Medicare (*p* < 0.001) (Table 1). Patients aged 50–64 years constituted the greatest proportion of the study population, particularly among those with commercial insurance (54.7%) and Medicaid (65.4%), whereas patients aged 65–75 years represented the majority of those insured with Medicare Advantage (65.7%) and Medicare (62.3%) (*p* < 0.001). Metropolitan areas had the highest proportion of patients across all payor categories: 82.7% in commercial, 79.9% in Medicare Advantage, 73.0% in Medicaid, and 75.9% in traditional Medicare (*p* < 0.001). Primary care providers (PCPs) ordered most tests, with the highest in Medicare (63.9%, *p* < 0.001). Most patients had a median household income based on zip code as $50,000 to $75,000 across all payor types: 37.8% in commercial, 44.7% in Medicare Advantage, 44.6% in Medicaid, and 41.6% in traditional Medicare (*p* < 0.001). Race, ethnicity, and preferred language were unknown for the majority of patients (78.32%, 69.16%, and 66.38%, respectively; Table 1).

**Table 1:** Sociodemographic characteristics of the study population

Characteristic	Commercial %, *N*	Medicare Advantage %, *N*	Medicaid %, *N*	Traditional Medicare %, *N*	Total %, *N*	*p* value
Study sample	100% (1,059,790)	100% (333,414)	100% (26,704)	100% (138,007)	100% (1,557,915)	
Sex						< 0.001
Female	57.69% (611,414)	58.50% (195,035)	59.20% (15,810)	57.40% (79,214)	57.86% (901,473)	
Male	42.31% (448,376)	41.50% (138,379)	40.80% (10,894)	42.60% (58,793)	42.14% (656,442)	
Age						< 0.001
45–49	30.63% (324,571)	4.97% (16,579)	24.69% (6593)	7.27% (10,033)	22.97% (357,776)	
50–64	54.66% (579,275)	16.91% (56,374)	65.42% (17,470)	16.49% (22,764)	43.38% (675,883)	
65–75	12.17% (128,994)	65.67% (218,965)	8.93% (2384)	62.34% (86,035)	28.01% (436,378)	
76–85	2.54% (26,950)	12.45% (41,496)	0.96% (257)	13.89% (19,175)	5.64% (87,878)	
Race						< 0.001
American Indian or Alaska Native	0.08% (837)	0.08% (272)	0.54% (143)	0.13% (177)	0.09% (1429)	
Asian Indian	0.98% (10,426)	0.63% (2097)	0.92% (247)	0.81% (1116)	0.89% (13,886)	
Black or African American	1.76% (18,686)	2.96% (9855)	4.10% (1096)	1.88% (2599)	2.07% (32,236)	
Other	0.06% (675)	0.05% (162)	0.15% (40)	0.07% (99)	0.06% (976)	
Unknown	78.68% (833,869)	78.22% (260,812)	73.72% (19,685)	76.62% (105,738)	78.32% (1,220,104)	
White	18.43% (195,297)	18.06% (60,216)	20.57% (5493)	20.49% (28,278)	18.57% (289,284)	
Ethnicity						< 0.001
Hispanic or Latino origin or descent	3.46% (36,708)	3.73% (12,435)	5.48% (1464)	2.55% (3524)	3.47% (54,131)	
Not of Hispanic, Latino/a, or Spanish origin	27.22% (288,432)	26.91% (89,731)	26.51% (7078)	29.42% (40,607)	27.33% (425,848)	
Other	0.03% (314)	0.03% (105)	0.04% (12)	0.03% (36)	0.03% (467)	
Unknown	69.29% (734,336)	69.33% (231,143)	67.97% (18,150)	68.00% (93,840)	69.16% (1,077,469)	
Preferred language						< 0.001
English	30.46% (322,823)	30.68% (102,278)	27.66% (7385)	30.24% (41,737)	30.44% (474,223)	
Other languages	0.43% (4586)	0.63% (2088)	2.18% (581)	0.81% (1119)	0.54% (8374)	
Spanish	2.59% (27,467)	3.12% (10,407)	4.53% (1211)	1.55% (2142)	2.65% (41,227)	
Unknown	66.51% (704,914)	65.58% (218,641)	65.63% (17,527)	67.39% (93,009)	66.38% (1,034,091)	
Urban/rural classification						< 0.001
Metropolitan	82.75% (876,956)	79.92% (266,449)	73.06% (19,511)	75.85% (104,678)	81.36% (1,267,594)	
Micropolitan	9.57% (101,408)	11.13% (37,115)	13.57% (3623)	12.49% (17,237)	10.23% (159,383)	
Rural	3.00% (31,827)	3.45% (11,515)	5.79% (1545)	4.84% (6685)	3.31% (51,572)	
Small town	4.68% (49,599)	5.50% (18,335)	7.58% (2025)	6.82% (9407)	5.09% (79,366)	
Provider specialty						< 0.001
GI specialist	1.51% (15,977)	2.57% (8568)	1.64% (439)	3.87% (5333)	1.95% (30,317)	
NP/PA	27.96% (296,110)	27.34% (91,137)	41.06% (10,958)	25.48% (35,144)	27.83% (433,349)	
OB/GYN	4.46% (47,223)	0.98% (3277)	2.14% (571)	1.29% (1774)	3.39% (52,845)	
Other	4.51% (47,807)	4.44% (14,787)	6.22% (1661)	5.51% (7608)	4.62% (71,863)	
PCP	61.56% (651,994)	64.67% (215,555)	48.93% (13,060)	63.86% (88,093)	62.21% (968,702)	
Median household income by zip code						< 0.001
$0 K–25 K	0.08% (821)	0.26% (875)	0.43% (116)	0.16% (225)	0.13% (2037)	
$25 K–50 K	8.85% (93,811)	16.67% (55,566)	20.61% (5503)	12.27% (16,939)	11.03% (171,819)	
$50 K–75 K	37.79% (400,490)	44.73% (149,141)	44.61% (11,912)	41.64% (57,473)	39.73% (619,016)	
$75 K–100 K	27.09% (287,145)	23.32% (77,741)	21.44% (5725)	24.71% (34,100)	25.98% (404,711)	
$100 K–150 K	21.64% (229,354)	13.38% (44,614)	11.41% (3048)	17.78% (24,535)	19.36% (301,551)	
$150 K–200 K	3.76% (39,865)	1.43% (4783)	1.24% (331)	2.86% (3947)	3.14% (48,926)	
> $200 K	0.78% (8304)	0.21% (694)	0.26% (69)	0.57% (788)	0.63% (9855)	

*GI*, gastroenterologist; *NP/PA*, nurse practitioner/physician assistant; *OB/GYN*, obstetrician/gynecologist; *PCP*, primary care physician

The overall adherence rate was 71.3% for the total population (Table 2). Adherence rates varied significantly across payor categories. Those covered through commercial health insurance plans had the highest adherence rate (72.3%), followed by Medicare Advantage (70.2%), traditional Medicare (69.9%), and Medicaid (52.0%) (*p* < 0.001). Females showed higher adherence rates than males across most payor types (Medicare Advantage: 70.4% vs. 69.8%, *p* < 0.001; Medicaid: 53.0% vs. 50.5%, *p* < 0.001; and traditional Medicare: 70.2% vs. 69.5%, *p* = 0.006) except for commercial (72.2% vs. 72.6%; *p* < 0.001). Patients aged 76–85 years had the highest adherence rates: 79.2% in commercial (*p* < 0.001), 76.5% in Medicare Advantage (*p* < 0.001), 54.9% in Medicaid (*p* = 0.232), and 76.3% in traditional Medicare (*p* < 0.001). Those residing in rural areas had the highest adherence rates across all payor types, except for Medicare: 73.2% in commercial insurance (*p* < 0.001), 71.3% in Medicare Advantage (*p* = 0.203), 54.1% in Medicaid (*p* < 0.001), and 70.7% in traditional Medicare (*p* < 0.001).

**Table 2:** Adherence rates in the study population

Characteristic	Commercial %, *N*	*p* value	Medicare Advantage %, *N*	*p* value	Medicaid %, *N*	*p* value	Traditional Medicare %, *N*	*p* value	Total %, *N*	*p* value
Adherence rate	72.34% (766,701)		70.16% (233,935)		51.96% (13,875)		69.94% (96,519)		71.32% (1,111,030)	< 0.001
Sex										0.1496
Female	72.15% (441,112)	< 0.001	70.43% (137,365)	< 0.001	52.97% (8374)	< 0.001	70.23% (55,634)	0.0057	71.27% (642,485)	
Male	72.62% (325,589)		69.79% (96,570)		50.50% (5501)		69.54% (40,885)		71.38% (468,545)	
Age group										< 0.001
45–49	71.69% (232,683)	< 0.001	56.51% (9368)	< 0.001	51.31% (3383)	0.2317	52.11% (5228)	< 0.001	70.06% (250,662)	
50–64	71.45% (413,877)		60.91% (34,337)		52.32% (9,141)		59.99% (13,656)		69.69% (471,011)	
65–75	76.60% (98,810)		72.38% (158,490)		50.76% (1210)		73.24% (63,011)		73.68% (321,521)	
76–85	79.15% (21,331)		76.49% (31,740)		54.86% (141)		76.27% (14,624)		77.19% (67,836)	
Race										< 0.001
American Indian or Alaska Native	58.06% (486)	< 0.001	63.24% (172)	< 0.001	47.55% (68)	< 0.001	66.10% (117)	< 0.001	58.99% (843)	
Asian Indian	71.06% (7409)		71.24% (1494)		60.32% (149)		70.70% (789)		70.87% (9841)	
Black or African American	63.08% (11,788)		60.44% (5956)		44.80% (491)		58.68% (1525)		61.30% (19,760)	
Other	55.70% (376)		58.02% (94)		45.00% (18)		50.51% (50)		55.12% (538)	
Unknown	73.27% (610,962)		70.96% (185,066)		52.62% (10,358)		70.40% (74,437)		72.19% (880,823)	
White	69.47% (135,680)		68.34% (41,153)		50.81% (2791)		69.32% (19,601)		68.87% (199,225)	
Ethnicity										< 0.001
Hispanic or Latino origin or descent	66.36% (24,358)	< 0.001	66.22% (8234)	< 0.001	51.02% (747)	0.0321	65.86% (2321)	< 0.001	65.88% (35,660)	
Not of Hispanic, Latino/a, or Spanish origin	71.86% (207,257)		69.71% (62,550)		50.58% (3580)		69.99% (28,420)		70.87% (301,807)	
Other	68.79% (216)		55.24% (58)		58.33% (7)		69.44% (25)		65.52% (306)	
Unknown	72.84% (534,870)		70.56% (163,093)		52.57% (9541)		70.07% (65,753)		71.77% (773,257)	
Preferred language										< 0.001
English	71.42% (230,567)	< 0.001	69.11% (70,687)	< 0.001	49.19% (3633)	< 0.001	69.64% (29,065)	0.1513	70.42% (333,952)	
Other languages	75.29% (3453)		77.11% (1610)		70.74% (411)		67.83% (759)		74.43% (6233)	
Spanish	70.20% (19,281)		73.02% (7599)		60.45% (732)		70.31% (1506)		70.63% (29,118)	
Unknown	72.83% (513,400)		70.45% (154,039)		51.91% (9099)		70.09% (65,189)		71.73% (741,727)	
Urban/rural classification										< 0.001
Metropolitan	72.23% (633,383)	< 0.001	70.09% (186,766)	0.0262	51.30% (10,010)	0.0059	69.64% (72,902)	0.0004	71.24% (903,061)	
Micropolitan	73.15% (74,181)		70.44% (26,143)		53.66% (1944)		70.96% (12,232)		71.84% (114,500)	
Rural	73.21% (23,302)		71.27% (8207)		54.05% (835)		70.67% (4724)		71.88% (37,068)	
Small town	72.25% (35,835)		69.92% (12,819)		53.63% (1086)		70.81% (6661)		71.06% (56,401)	
Provider specialty										< 0.001
GI specialist	82.54% (13,187)	< 0.001	81.31% (6967)	< 0.001	59.23% (260)	0.0057	80.29% (4282)	< 0.001	81.46% (24,696)	
NP/PA	69.69% (206,349)		66.50% (60,609)		51.13% (5603)		66.15% (23,248)		68.26% (295,809)	
OB/GYN	72.20% (34,096)		70.34% (2305)		54.12% (309)		70.18% (1245)		71.82% (37,955)	
Other	72.57% (34,694)		68.28% (10,096)		51.41% (854)		69.83% (5313)		70.91% (50,957)	
PCP	73.29% (477,869)		71.39% (153,890)		52.40% (6843)		70.83% (62,392)		72.36% (700,994)	
Median household income by zip code										< 0.001
$0 K–25 K	63.58% (522)	< 0.001	60.00% (525)	< 0.001	41.38% (48)	< 0.001	54.22% (122)	< 0.001	59.74% (1217)	
$25 K–50 K	68.19% (63,967)		64.38% (35,771)		48.23% (2654)		64.37% (10,903)		65.94% (113,295)	
$50 K–75 K	71.26% (285,383)		69.88% (104,222)		51.54% (6139)		69.10% (39,716)		70.35% (435,460)	
$75 K–100 K	73.21% (210,215)		72.39% (56,273)		53.78% (3079)		71.18% (24,274)		72.61% (293,841)	
$100 K–150 K	74.25% (170,304)		74.01% (33,017)		56.33% (1717)		73.16% (17,949)		73.95% (222,987)	
$150 K–200 K	75.19% (29,974)		74.89% (3582)		58.01% (192)		74.74% (2950)		75.01% (36,698)	
> $200 K	76.30% (6336)		78.53% (545)		66.67% (46)		76.78% (605)		76.43% (7532)	

*GI*, gastroenterologist; *NP/PA*, nurse practitioner/physician assistant; *OB/GYN*, obstetrician/gynecologist; *PCP*, primary care physician

Across all payor types, the adherence rates were highest for those whose tests were ordered by GI specialists: 82.5% in commercial insurance (*p* < 0.001), 81.3% in Medicare Advantage (*p* < 0.001), 59.2% in Medicaid (*p* = 0.0057), and 80.3% in traditional Medicare (*p* < 0.001). Adherence rates increased with higher income brackets across all payor types: 76.3% for those earning over $200,000 in commercial insurance (*p* < 0.001), 78.5% in Medicare Advantage (*p* < 0.001), 66.7% in Medicaid (*p* < 0.001), and 76.8% in traditional Medicare (*p* < 0.001).

The average time to adherence varied across demographic subgroups, with an overall mean of 26.4 days (Table 3). Patients with commercial insurance had a mean time to adherence of 27.2 days, compared to 24.4 days for Medicare Advantage, 26.3 days for Medicaid, and 24.7 days for Medicare. Sex differences were notable, with females generally taking longer than males across most payor types, except for Medicaid, where the difference was not statistically significant (*p* value = 0.6732). The 76–85 age group showed the shortest time to adherence overall, with a mean of 20.3 days (*p* < 0.001). Rural residents had the longest average times to adherence across all payor types, with an overall mean of 27.0 days (*p* < 0.001). GI specialists had the shortest overall adherence times, with a mean of 21.9 days (*p* < 0.001). Adherence times varied by income across different payor types: for commercial and Medicaid plans, individuals with an income between $25 K–$50 K had the shortest time to adherence, with averages of 25.9 days (*p* < 0.001) and 24.9 days (*p* = 0.0037), respectively. In contrast, for Medicare Advantage and Medicare, the shortest time to adherence was observed in the $150 K–$200 K income bracket, averaging 22.8 days (*p* < 0.001) and 23.8 days (*p* = 0.0274), respectively.

**Table 3:** Time to adherence in the study population

Characteristic	Commercial			Medicare Advantage			Medicaid			Traditional Medicare			Total		
Time to adherence (*n* days)	Mean	SD	*p* value	Mean	SD	*p* value	Mean	SD	*p* value	Mean	SD	*p* value	Mean	SD	*p* value
Overall	27.2	42.8		24.4	37.8		26.3	39.0		24.7	39		26.4	41.5	
Sex															
Female	27.9	43.7	< 0.001	25.1	38.5	< 0.001	26.2	38.4	0.6732	25.3	39.8	< 0.001	27.1	42.2	< 0.001
Male	26.2	41.7		23.5	36.8		26.5	40.0		23.9	37.8		25.5	40.4	
Age group															
45–49	29.3	46.7	< 0.001	29.1	43.9	< 0.001	26.6	39.5	0.6370	31.1	45.3	< 0.001	29.3	46.4	< 0.001
50–64	27.3	42.7		28.3	43.8		26.4	38.9		30.1	45.9		27.4	42.8	
65–75	23.1	35.4		24.1	37.2		25.2	39.5		24.0	38.1		23.8	36.9	
76–85	19.9	29.4		20.4	30.7		24.0	35.6		20.7	31.7		20.3	30.6	
Race															
American Indian or Alaska Native	32.3	52.2	< 0.001	29.9	47.2	< 0.001	27.9	41.5	0.6551	35.6	52.1	< 0.001	31.9	50.4	< 0.001
Asian Indian	26.5	42.3		23.6	38.7		21.5	25.9		22.5	30.9		25.6	40.8	
Black or African American	26.3	41.6		26.9	43.3		26.2	31.5		27.9	43.6		26.6	42.1	
Other	33.3	53.3		27.5	36.7		27.9	41.5		27.4	19.8		31.5	48.1	
Unknown	27.1	42.7		24.4	37.5		26.2	39.3		24.7	38.8		26.3	41.3	
White	27.4	43.7		24.2	38.2		27.0	39.9		24.8	39.3		26.5	42.2	
Ethnicity															
Hispanic or Latino origin or descent	27.3	41.0	0.7590	26.6	37.9	< 0.001	26.3	40.3	0.1453	26.5	38.3	0.0274	27.0	40.1	0.014
Not of Hispanic, Latino/a, or Spanish origin	27.1	43.4		24.4	38.9		27.6	42.0		24.8	39.6		26.4	42.2	
Other	30.1	47.6		21.7	17.9		28.1	16.5		40.8	70.3		29.3	45.5	
Unknown	27.2	42.7		24.3	37.4		25.8	37.8		24.7	38.7		26.4	41.3	
Preferred language															
English	27.5	44.0	< 0.001	24.3	37.9	< 0.001	27.8	43.2	0.0042	25.2	40.5	0.0016	26.6	42.5	< 0.001
Other languages	21.6	30.4		22.2	35.3		22.9	38.1		26.9	44.9		22.5	34.3	
Spanish	27.0	38.4		27.6	39.0		23.3	33.0		27.1	37.4		27.0	38.4	
Unknown	27.1	42.6		24.4	37.7		26.1	37.7		24.5	38.2		26.3	41.2	
Urban/rural classification															
Metropolitan	27.3	43.4	< 0.001	24.6	38.2	< 0.001	26.1	39.6	0.5437	24.8	39.7	0.0113	26.5	42.0	< 0.001
Micropolitan	26.3	40.2		23.4	34.9		27.1	39.8		24.2	36.7		25.5	38.7	
Rural	27.7	41.7		25.5	39.1		27.6	38.2		26.1	39.6		27.0	40.8	
Small town	26.1	39.7		24.0	37.1		25.8	33.1		24.0	34.0		25.4	38.4	
Provider specialty															
GI specialist	22.2	35.8	< 0.001	20.6	33.3	< 0.001	30.4	43.9	0.1277	22.6	34.9	0.0015	21.9	35.1	< 0.001
NP/PA	27.1	41.7		25.0	38.0		26.1	39.0		25.2	39.1		26.5	40.7	
OB/GYN	28.9	46.4		25.5	43.8		23.7	31.4		24.7	36.0		28.6	45.9	
Other	26.9	41.4		24.6	37.1		28.5	45.3		25.4	38.7		26.3	40.4	
PCP	27.2	43.4		24.4	37.9		26.2	38.4		24.7	39.3		26.4	41.8	
Median household income by zip code															
$0 K–25 K	31.7	46.3	< 0.001	27.5	38.3	< 0.001	25.4	21.1	0.3267	28.8	39.2	0.3000	29.3	41.6	< 0.001
$25 K–50 K	25.9	39.1		25.1	38.3		24.9	36.5		25.4	39.1		25.6	38.8	
$50 K–75 K	26.6	41.2		24.5	37.5		26.2	37.8		24.6	37.6		25.9	40.0	
$75 K–100 K	27.5	43.7		24.3	37.5		27.5	42.8		24.8	39.4		26.7	42.2	
$100 K–150 K	28.0	45.2		24.0	38.8		26.6	39.8		24.7	41.0		27.1	44.0	
$150 K–200 K	27.9	45.6		22.8	37.6		26.2	41.7		23.8	39.8		27.1	44.4	
> $200 K	27.7	45.4		24.7	40.4		30.4	51.9		25.4	43.9		27.3	45.0	

*GI*, gastroenterologist; *NP/PA*, nurse practitioner/physician assistant; *OB/GYN*, obstetrician/gynecologist; *PCP*, primary care physician

In the logistic regression analysis (Table 4), several factors were associated with adherence. Males had similar adherence odds compared to females (OR: 1.00, *p* = 0.092). Compared to the 45–49 age group, those aged 50–64 (OR: 1.01, *p* = 0.003), 65–75 (OR: 1.47, *p* < 0.001), and 76–85 (OR: 1.69, *p* < 0.001) had significantly higher odds of adherence. Rural (OR: 1.19, *p* < 0.001), micropolitan (OR: 1.17, *p* < 0.001), and small-town residents (OR: 1.16, *p* < 0.001) had higher odds of adherence compared to those in metropolitan areas. Those with Medicare Advantage (OR: 0.72, *p* < 0.001), Medicaid (OR: 0.45, *p* < 0.001), and Traditional Medicare (OR: 0.69, *p* < 0.001) had lower odds of adherence compared to those with commercial insurance. NP/PAs (OR: 0.53, *p* < 0.001), OB/GYNs (OR: 0.62, *p* < 0.001), other provider types (OR: 0.59, *p* < 0.001), and PCPs (OR: 0.62, *p* < 0.001) all had significantly lower odds of adherence compared to GI specialists. Compared to the $0 K–25 K income bracket, those in the $25 K–50 K bracket had significantly higher odds of adherence (OR: 1.23, *p* < 0.001). Similarly, individuals in the $50 K–75 K (OR: 1.45, *p* < 0.001), $75 K–100 K (OR: 1.62, *p* < 0.001), $100 K–150 K (OR: 1.70, *p* < 0.001), $150 K–200 K (OR: 1.76, *p* < 0.001), and > $200 K (OR: 1.86, *p* < 0.001) brackets all showed progressively higher odds of adherence.

**Table 4:** Logistic regression—factors associated with adherence in the study population

Characteristic	OR	95%CI	*p* value
Sex			
Female	Ref	Ref	Ref
Male	1.00	0.99–1.01	0.0920
Age			
45–49	Ref	Ref	Ref
50–64	1.01	1.00–1.02	0.0030
65–75	1.47	1.45–1.49	< 0.001
76–85	1.69	1.66–1.72	< 0.001
Race			
American Indian or Alaska Native	Ref	Ref	Ref
Asian Indian	1.35	1.20–1.51	< 0.001
Black or African American	1.08	0.96–1.20	0.1860
Other	0.78	0.66–0.92	0.0040
Unknown	1.66	1.49–1.84	< 0.001
White	1.36	1.22–1.51	< 0.001
Ethnicity			
Hispanic or Latino origin or descent	Ref	Ref	Ref
Not of Hispanic, Latino/a, or Spanish origin	1.33	1.31–1.36	< 0.001
Other	1.02	0.84–1.24	0.8340
Unknown	1.24	1.22–1.27	< 0.001
Preferred language			
English	Ref	Ref	Ref
Other languages	1.26	1.20–1.33	< 0.001
Spanish	1.19	1.16–1.22	< 0.001
Unknown	1.08	1.07–1.09	< 0.001
Urban/rural classification			
Metropolitan	Ref	Ref	Ref
Micropolitan	1.17	1.15–1.18	< 0.001
Rural	1.19	1.17–1.22	< 0.001
Small town	1.16	1.14–1.17	< 0.001
Payor type			
Commercial	Ref	Ref	Ref
Medicare Advantage	0.72	0.71–0.72	< 0.001
Medicaid	0.45	0.44–0.46	< 0.001
Traditional Medicare	0.69	0.68–0.70	< 0.001
Provider specialty			
GI specialist	Ref	Ref	Ref
NP/PA	0.53	0.52–0.55	< 0.001
OB/GYN	0.62	0.60–0.64	< 0.001
Other	0.59	0.57–0.61	< 0.001
PCP	0.62	0.60–0.64	< 0.001
Median household income by zip code			
$0 K–25 K	Ref	Ref	Ref
$25 K–50 K	1.23	1.12–1.35	< 0.001
$50 K–75 K	1.45	1.33–1.59	< 0.001
$75 K–100 K	1.62	1.48–1.77	< 0.001
$100 K–150 K	1.70	1.56–1.87	< 0.001
$150 K–200 K	1.76	1.60–1.93	< 0.001
> $200 K	1.86	1.68–2.06	< 0.001

*GI*, gastroenterologist; *NP/PA*, nurse practitioner/physician assistant; *OB/GYN*, obstetrician/gynecologist; *PCP*, primary care physician

## Discussion

This study provides updated mt-sDNA CRC screening adherence among a diverse cohort of over 1.5 million patients insured by four of the largest payors in the USA. With an overall adherence rate of 71.3%, the findings underscore the effectiveness of mt-sDNA testing in facilitating CRC screening and promoting early detection. The substantial adherence rate reflects the test’s potential to enhance screening uptake and, consequently, contribute to reducing CRC-related mortality rates. The large-scale data set revealed significant variations in adherence across different payor types and other subgroups.

The highest adherence rate was 72.3% for individuals with commercial insurance, while those with Medicaid had a lower rate of 52.0%. This disparity reflects differences in healthcare access and screening behaviors among Medicaid recipients. Although mt-sDNA yields benefit through excellent coverage by most payors [15], the lower adherence in this group may be linked to factors associated with socioeconomic challenges, such as lower health literacy, less frequent healthcare interactions, or limited awareness of screening benefits [16–19]. To effectively address these challenges, further research is needed to better understand these factors and develop tailored interventions to improve adherence in lower-income populations.

Adherence varied by age, with the highest rates observed in older age groups, particularly those aged 76–85 years, and lower rates in younger cohorts. This variation is possibly due to more frequent utilization of healthcare services by older age groups for managing existing chronic conditions, where providers are more proactive in both recommending and ensuring adherence to screenings [20–22]. Logistic regression results showed significantly higher odds of adherence for the older age group than the youngest age group (patients aged 45–49 years). Younger populations had the lowest odds of adherence, which may be due to differing personal and professional stages and unique barriers to screening compared to individuals older individuals [23]. Nevertheless, due to the observed increases in CRC incidence in younger age groups, it is essential to boost screening interventions for this population [4]. Convenient, noninvasive, at-home screening tests such as mt-sDNA may be particularly well-suited for closing the screening gap among younger individuals with demanding schedules [24].

Disparities in adherence rates by rural/urban status were evident, with rural areas reporting slightly higher adherence rates than metropolitan areas. Logistic regression confirmed that individuals in rural areas have significantly higher adherence odds than those from metropolitan areas (OR: 1.19). This counterintuitive finding is consistent with the earlier study on mt-sDNA adherence and may reflect more personalized care and community support in rural settings, which could enhance preventive care efforts [14, 25]. However, given that the rural population constituted only 3% of the overall study population, these geographic differences may limit the generalizability of the findings.

Provider specialty was associated with adherence to mt-sDNA testing, with gastroenterologists (GIs) showing significantly higher adherence rates. This is likely due to factors such as the strength of their recommendations, their expertise in colorectal cancer prevention, patient attitudes toward follow-up care, and established relationships with patients [14]. However, GIs ordered a relatively small proportion of mt-sDNA tests, while primary care providers (PCPs) ordered the majority. Despite this, tests ordered by PCPs were associated with significantly lower adherence rates than those ordered by GIs. This discrepancy highlights a key opportunity to improve adherence to tests ordered by PCPs. To address this, strategies such as training programs for PCPs could enhance their understanding of mt-sDNA testing, improve communication with patients, and reinforce the importance of follow-up care [26]. Additionally, integrating decision support tools into electronic health records (EHRs) could assist PCPs by identifying eligible patients, tracking screening progress, and prompting timely follow-up [26–28]. Focusing on these approaches may help close the adherence gap between tests ordered by GIs and PCPs, ultimately improving the overall effectiveness of CRC screening and ensuring better patient outcomes.

Socioeconomic factors were also associated with adherence, with patients from higher-income brackets demonstrating higher adherence rates. This pattern is consistent with research indicating that individuals with higher socioeconomic statuses have better access to health care resources and are more likely to engage in preventive screenings [29–31]. Logistic regression results confirmed that patients in higher income brackets had significantly higher adherence odds than those earning $0–25 K. Like the discussion on Medicaid above, lower adherence among lower-income individuals—despite mt-sDNA being covered by Medicare and most major insurers—may not be directly related to cost but rather influenced by other underlying factors. Further exploration into these factors is necessary to develop targeted interventions addressing specific adherence barriers in lower-income populations.

This study has several acknowledged limitations. Firstly, data on race, ethnicity, and preferred language were minimal, as patients were not required to provide this information as part of the mt-sDNA test order. This constraint hindered a comprehensive analysis of racial and ethnic disparities in adherence rates. Additionally, the analysis was restricted to only those patient and provider variables that were available within the existing laboratory data at the time. Moreover, CRC risk status for mt-sDNA testing was based on providers’ clinical evaluations. With limited data available to verify these assessments, off-label test usage cannot be ruled out. Furthermore, the study focused on cross-sectional adherence over only a 1-year period without evaluating long-term adherence. This relatively short timeframe may not capture variations in adherence that could emerge over extended periods. The lack of a comparative analysis with alternative screening methods limited context on relative adherence rates. Nevertheless, a previous analysis by Miller-Wilson et al. in 2021 reported a 66.8% overall adherence rate for mt-sDNA, while this study found higher overall (71.3%) and subgroup-specific adherence rates over a more recent time period [14]. Lastly, potential biases related to the distribution of test kits or variations in patient follow-up practices across different healthcare settings were not considered. Future research should aim to include a more diverse range of demographic and socioeconomic factors, assess adherence over extended periods, and explore comparative adherence across multiple screening modalities.

## Conclusions

The current study highlights a robust adherence rate of 71.3% to mt-sDNA testing, particularly among individuals with commercial insurance, older adults, gastroenterology patients, higher-income individuals, and rural residents. While engagement is strong overall, there is an opportunity to enhance participation, especially within the Medicaid population. Future research should focus on longitudinal studies to assess adherence over time and explore relationships with socioeconomic status, race/ethnicity, and other factors. Addressing disparities through tailored outreach and support strategies will be crucial for maximizing the benefits of mt-sDNA and achieving national CRC screening goals.

## Supplementary Information

Below is the link to the electronic supplementary material.

Supplementary file1 (DOCX 34 KB)

## Data Availability

The study data are available upon reasonable request, subject to applicable regulations and company policies.
